# Optimized Sodium-Enhanced MALDI‑2 Enables Multimodal Imaging of Triacylglycerols

**DOI:** 10.1021/jasms.6c00227

**Published:** 2026-08-25

**Authors:** Kameron R. Molloy, Cody R. Marshall, Martin Dufresne, Lukasz G. Migas, Matthew S. Schrag, Raf Van de Plas, Jeffrey M. Spraggins

**Affiliations:** † Department of Chemistry, 5718 Vanderbilt University, Nashville, Tennessee 37235, United States; ‡ Mass Spectrometry Research Center, Vanderbilt University, Nashville, Tennessee 37235, United States; § Chemical and Physical Biology Program, Vanderbilt University School of Medicine, Nashville, Tennessee 37240, United States; ∥ Department of Cell and Developmental Biology, Vanderbilt University, Nashville, Tennessee 37232, United States; ⊥ Delft Center for Systems and Control, 2860 Delft University of Technology, 2628 Delft, Netherlands; # Department of Neurology, 12327 Vanderbilt University School of Medicine, Nashville, Tennessee 37232, United States; ¶ Vanderbilt Brain Institute, Vanderbilt University, Nashville, Tennessee 37240, United States; ∇ Vanderbilt Memory and Alzheimer’s Center, Vanderbilt University Medical Center, Nashville, Tennessee 37232, United States; ○ Department of Biochemistry, Vanderbilt University, Nashville, Tennessee 37205, United States; ⧫ Department of Pathology, Microbiology and Immunology, Vanderbilt University Medical Center, Nashville, Tennessee 37205, United States

**Keywords:** TAGs, neutral lipids, Alzheimer's disease, cerebral amyloid angiopathy, spatial lipidomics, multimodal imaging, tissue mapping, sensitivity, MALDI-2

## Abstract

Triacylglycerols (TAGs) are essential neutral lipids with critical roles in cellular function and disease. However, spatial characterization of these chemical species remains difficult because neutral molecules often exhibit low ionization efficiency with common mass spectrometry-based imaging strategies, such as matrix-assisted laser desorption/ionization imaging mass spectrometry (MALDI IMS). Here, we assess how ammonium formate (AmF) or sodium carbonate buffer solution (CBS) tissue washes, combined with varying primary laser power, affect TAG sensitivity with and without MALDI-2 postionization. We found that Na^+^ doping using Na^+^ CBS washes combined with MALDI-2 at 31% total laser power was optimal for both signal intensity and molecular coverage. Additionally, Na^+^ CBS-washed tissues enabled MALDI-2 to be performed using lower laser powers, making this method compatible with sequential microscopy experiments as part of multimodal workflows. Applying this strategy to diseased brain tissue enabled spatial mapping of TAGs linked to Alzheimer’s disease. This approach balances sensitivity and tissue preservation, providing a robust platform for multimodal spatial lipidomic studies across diverse tissues and disease contexts.

## Graphical abstract

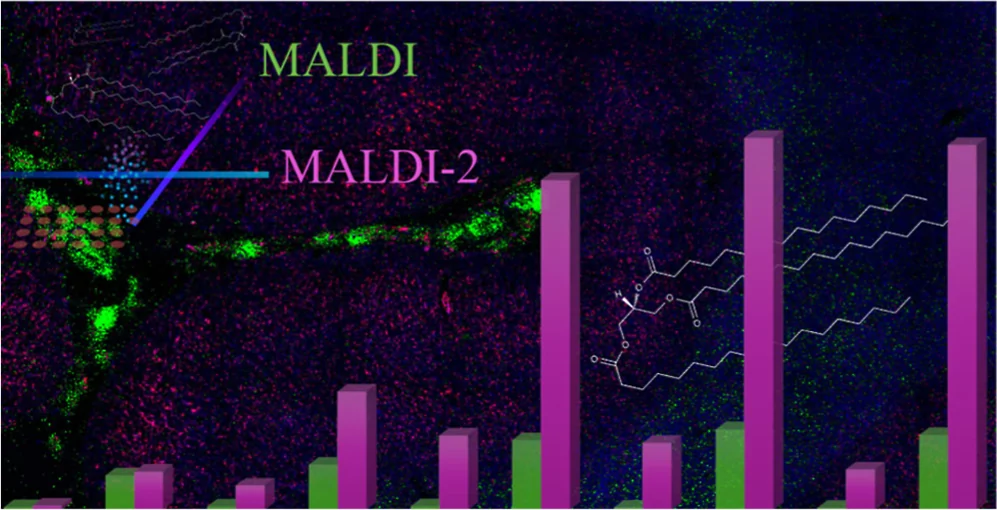

## Introduction

Triacylglycerols (TAGs) are neutral lipids composed of a glycerol backbone and three fatty acid chains. These biomolecules are the main form of long-term, dense energy storage in the human body. Emerging evidence has linked TAG levels to the development and progression of neurological disorders, such as Alzheimer’s disease.^1^ While bulk lipidomic analyses show elevated levels of TAGs in the brain tissue of Alzheimer’s patients, few studies have mapped their localization in tissue microenvironments.^1,2^ Understanding complex neuropathologies requires multimodal, spatially driven analyses that use complementary imaging techniques to capture molecular and structural heterogeneity (e.g., histological staining, immunofluorescence microscopy, spatial transcriptomics, etc.). Matrix-assisted laser desorption/ionization imaging mass spectrometry (MALDI IMS) has emerged as a key tool for spatial lipidomics, offering robust biomolecule detection and multiplexing capacity at cellular resolution. However, neutral molecules like TAGs have poor ionization efficiency by MALDI and are subject to ion suppression by highly abundant phospholipids.^3^ Several strategies have been developed to overcome this challenge, including optimized sample preparation methods (e.g., salt doping) and postionization techniques (MALDI-2).^4–6^ While successful in enhancing neutral lipid sensitivity, these approaches have yet to be assessed for their compatibility with multimodal imaging workflows.

Here, we evaluate the impact of combined salt doping and MALDI-2 on TAG sensitivity in a multimodal imaging context. Three experimental parameters were varied: tissue washing protocol, ionization method, and primary laser power. Tissue quality post-IMS-analysis is also examined to gauge compatibility with sequential multimodal measurement. TAG sensitivity was enhanced with salt doping and MALDI-2, both separately and combined. The efficacy of both improved with higher primary laser powers (i.e., the power of the laser directed at the sample to facilitate MALDI). These findings were used to inform experimental design for a case study mapping TAGs in vascular regions of Alzheimer’s brain tissue with cerebral amyloid angiopathy (CAA). By integrating immunofluorescence and staining microscopy post-MALDI IMS, we established salt doping as a robust approach compatible with multimodal spatial omics studies.

## Experimental Section

A multitissue homogenate (rat brain, rat liver, rabbit testes, and rabbit adrenal gland) and Alzheimer’s diseased and CAA-positive human brain tissue (IRB protocol #180287) were sectioned and thaw-mounted onto ITO-coated glass slides before washing in either ammonium formate (AmF) or Na^+^ carbonate buffer solution (CBS).^7^ 2′,5′-dihydroxyacetophenone acid (DHA, 1.6 μg/mm^2^ or 3.1 ± 0.2 μg/mm^2^) was sublimated onto the slides, then positive ion mode MALDI IMS was performed on a timsTOF fleX MALDI-2 mass spectrometer equipped with a SRIG mobility funnel (Bruker Daltonik, Bremen, Germany). Both Na^+^ CBS and AmF-washed homogenate sections were imaged in triplicate with and without MALDI-2 at five different primary laser powers, namely with total laser power (TLP) at 21%, 26%, 31%, 36%, and 41%. The TLP calculation is provided in the Supporting Information. Following homogenate evaluation, the optimal parameters were applied to Alzheimer’s brain tissue, which was washed with Na^+^ CBS then imaged at 15 μm pitch with MALDI-2 at 31% TLP.

Microscopy images were collected after IMS measurement. First, homogenate sections were stained with hematoxylin and eosin (H&E). Then, brain tissues were stained with thiazine red and primary antibodies for Collagen IV and αSMA, and then coverslipped. After immunofluorescence imaging, coverslips were removed to H&E stain the brain tissues before performing brightfield microscopy. A Zeiss AxioScan.Z1 fluorescence slide scanner (Carl Zeiss Microscopy GmbH, Oberkochen, Germany) was used to collect all fluorescence and brightfield microscopy images.

Ion images were visualized with SCiLS (Bruker Daltonik, Bremen, Germany). Data were preprocessed using in-house developed software that performs peak alignment, recalibration, and total ion current normalization before intensity values and spectra were extracted.^8^ Average spectra were then visualized using mMass (version 5.5.0).^9^ IMS peak annotations and putative identifications were made using accurate mass matching with a 5 ppm mass error threshold compared to the LIPIDMAPS database and verified using previously reported LC–MS/MS.^5,10^ While these annotations align with previously reported LC–MS/MS findings in rat brain and rabbit adrenal tissues, it should be noted that the lack of on-tissue MS/MS or orthogonal separations limits our ability to differentiate between isomeric or isobaric species. Any annotated lipid that was not detected (S/*N* > 3) in all three replicates was excluded from further analysis. A curated list of TAG species was used to evaluate TAG detection ([M + H]^+^ and [M + Na]^+^ adducts only) across imaging experiments. Since the smallest data set contained 1931 pixels, all other data sets were subsampled to 1931 pixels. The number of detected TAGs was counted for each data set, and corresponding intensities were extracted. Expanded methods and mass errors for each annotation are available in the Supporting Information.

## Results

### TAG Sensitivity Enhancement

Using a multitissue homogenate, we examined how varying the primary laser power influenced TAG sensitivity and tested the effects of MALDI-2 and two salt washes. The number and intensity of annotated TAGs from a curated list (Figure 1a) were evaluated across conditions. Figure 1b summarizes the average intensity of TAGs detected for each combination of wash, laser power, and ionization method. In every case, MALDI-2 (purple) enhanced TAG sensitivity relative to MALDI alone (green), while Na^+^ CBS washing consistently yielded higher TAG intensities than AmF for both ionization methods. Focusing specifically on total laser power for Na^+^ CBS-washed samples using MALDI-2, the highest average intensity was observed at 36% TLP. Notably, similar intensities were observed at 31%, 36%, and 41% TLP, suggesting that robust TAG detection is achieved at 31% TLP, with higher laser powers yielding diminishing returns in sensitivity (Figure 1b).

**Figure 1:**
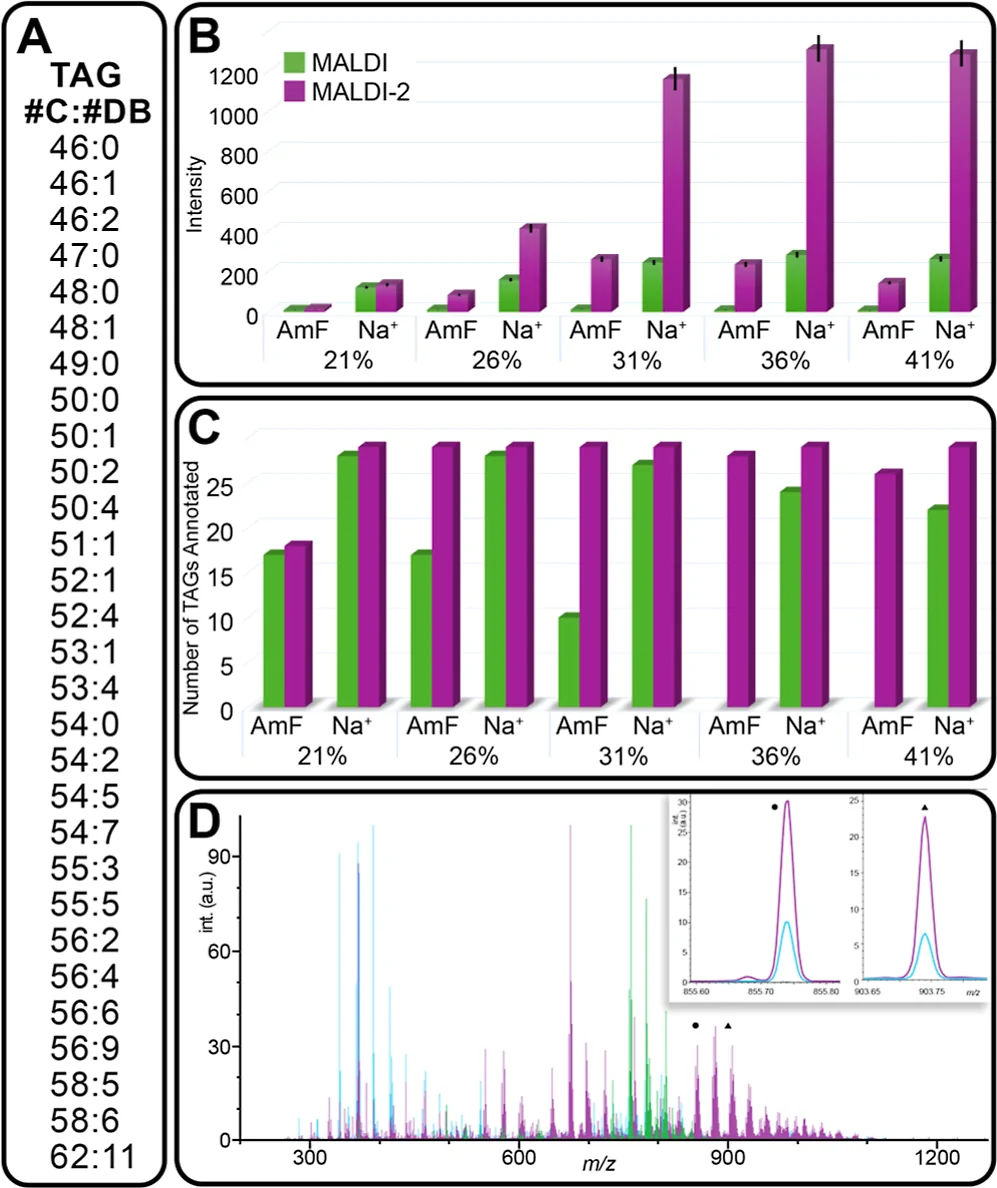
A complete list of TAG species detected across all three replicate experiments (A), which was used for comparison of TAG intensities across data sets and conditions (B), and the number of annotated TAGs per data set with a signal-to-noise ratio above 3 (C). For simplicity, only the [M + H]^+^ and [M + Na]^+^ were considered (B & C). The average spectrum from three data sets is depicted with zoomed views of TAG 50:1 (left) and TAG 54:4 (right) (D). AmF washed samples at 21% TLP is shown in green, Na^+^ CBS-washed samples at 21% TLP in blue, and Na^+^ CBS-washed samples with MALDI-2 at 31% TLP in purple.

Figure 1c highlights the number of TAGs annotated (S/*N* > 3) for each parameter combination. With AmF-washed samples, TAG coverage was highly dependent on the ionization method. MALDI-2 (purple) enabled detection of at least 26 of the 29 TAGs at total laser powers above 21%. Conventional MALDI (green) combined with AmF washes detected no more than 17 TAG annotations, with fewer TAGs detected at total laser powers above 26% and none detected at 36% or 41% TLP. These experiments clearly demonstrate that for AmF-washed tissues, MALDI-2 performance benefits from higher energy input from the primary laser, aligning with previous findings.^11^ In contrast, Na^+^ CBS doping resulted in consistently high TAG coverage (≥22 annotations) regardless of the ionization technique. However, it is important to consider that previous findings suggest this method enhances the sensitivity of other neutral lipid classes.^5,6^ Consequently, these additional neutral lipids, with the phospholipid population that remains present, may compete during ionization and suppress TAG species. Nevertheless, comparable numbers of TAGs were detected by both MALDI and MALDI-2 across all total laser powers, with only slight losses observed with conventional MALDI at total laser powers above 31%. These results suggest that Na^+^ CBS doping with MALDI-2 at 31% TLP (Figure 1d, purple) offers an optimal balance between signal intensity and species coverage. The spectral differences in Figure 1d reinforce this observation. Many new peaks were detected with Na^+^ CBS doping (blue and purple) compared with the AmF-washed homogenate (green). Moreover, the zoomed spectra of two TAG species make it evident that MALDI-2 (purple) increases TAG sensitivity when compared to the same conditions without MALDI-2 (blue).

Importantly, for Na^+^ CBS-washed MALDI-2 experiments, all annotated TAG lipids were detected even at lower total laser power, indicating that laser power affects intensity, but not necessarily molecular coverage. This highlights the practical considerations made when optimizing methods for molecular imaging experiments. Higher laser powers (TLP ≥31%) will maximize signal-to-noise ratios, which may be needed to produce high-quality molecular images, particularly for low-intensity species. However, for certain experiments, lower laser powers may be preferred to maintain the integrity of more delicate tissues. These observations highlight the flexibility of combining Na^+^ CBS doping with MALDI-2 to tailor MALDI IMS experiments to maximize sensitivity and molecular coverage while ensuring compatibility with multimodal workflows.

### Multimodal Compatibility

Multimodal imaging relies on the preservation of the tissue throughout the workflow, requiring a careful balance of laser power, wash protocol, and matrix concentration. Poor tissue preservation can inhibit complementary same-section microscopy assays, prohibiting cellular-level analyses. The importance of these factors for multimodal studies is made clear in Figure 2a, which shows resulting primary laser ablation craters in a multitissue homogenate. The AmF washed homogenate showed no obvious tissue damage with ≤26% TLP (top), while the Na^+^ CBS washed homogenate with the same matrix concentration showed damage at total laser powers above 21% TLP (middle). As shown previously, incorporation of salt washes impacts matrix crystallization,^6,12^ which can affect how the laser interacts with the tissue. A Na^+^ CBS-washed sample with twice the matrix concentration was also evaluated (bottom). Increased matrix concentration substantially improved tissue preservation, allowing ≤31% TLP to be used with no visible ablation craters. Importantly, when the tissue homogenate was evaluated with increased matrix concentration, TAG signal intensity was reduced, but all the TAG annotations were retained (Figure S4). Thus, Na^+^ CBS washing combined with increased matrix concentration and 31% TLP provides a practical compromise for multimodal analyses by facilitating subsequent microscopy assays while preserving sufficient TAG sensitivity.

**Figure 2:**
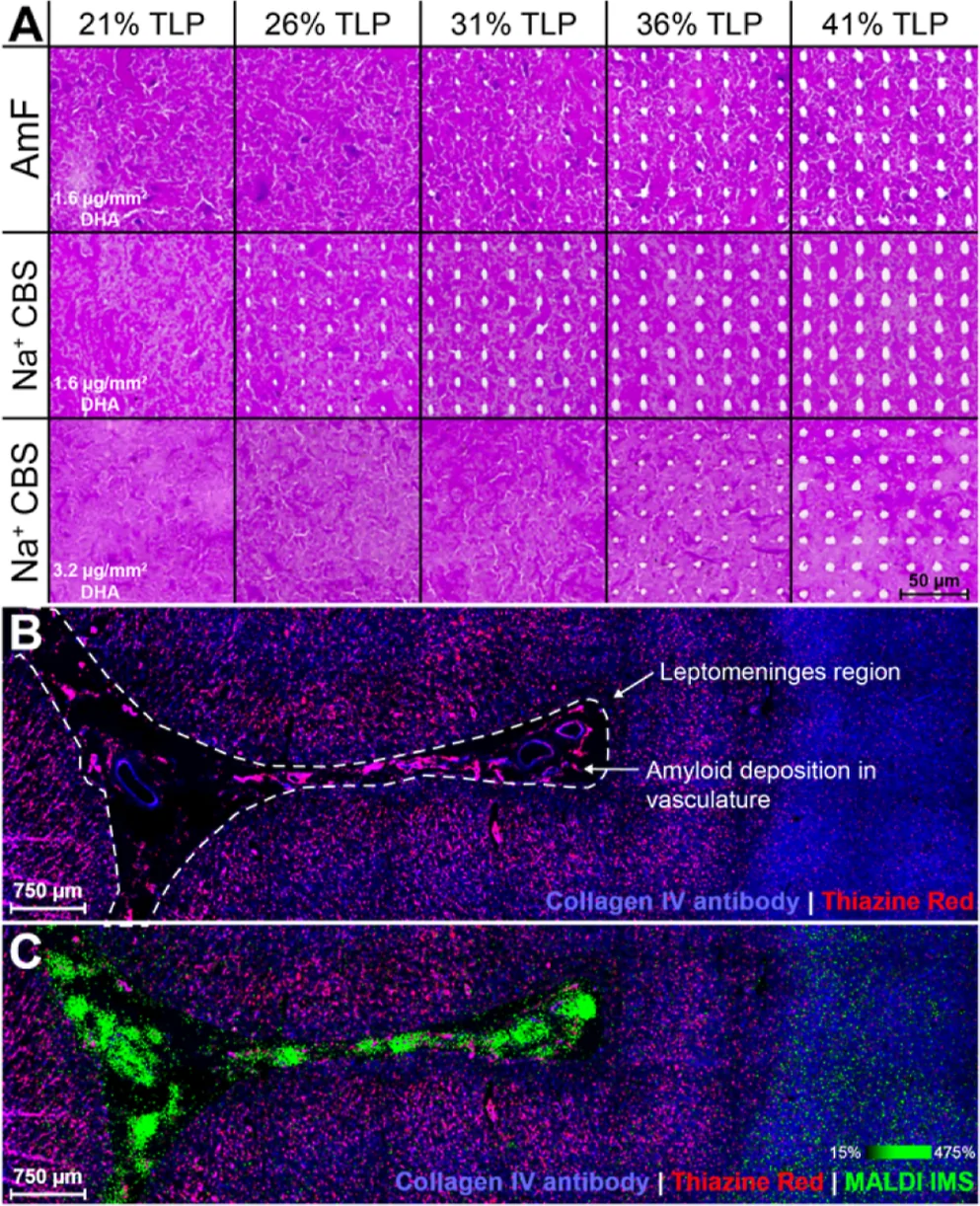
Homogenate H&E stains reveal ablation craters from the primary laser (A) when washed with AmF (top), Na^+^ CBS (middle) and Na^+^ CBS with increased matrix concentration (bottom). Using optimal parameters (Na^+^ CBS wash, 31% TLP, and 3.2 μg/mm^2^ DHA), multimodal imaging data acquired from human brain tissue collected from an Alzheimer’s diseased and CAA-impacted donor. Immunofluorescence microscopy (B) was collected following MALDI IMS (C). The dotted line in (B) highlights the leptomeninges region of the tissue, with Collagen IV (blue) marking vascular regions and thiazine red (red) highlighting amyloid deposits. The ion image of TAG 50:0, shown in green, is coregistered and overlaid with the associated microscopy data in (C).

### Alzheimer Disease Case Study

The efficacy of Na^+^ CBS doping and MALDI-2 was then evaluated using human brain tissue exhibiting Alzheimer’s disease and cerebral amyloid angiopathy (CAA) pathologies. Multimodal analyses are essential for mapping molecular distributions to disease-associated tissue microenvironments in situ. Given the small spatial scale of many Alzheimer’s disease features, acquisition from the same tissue section is needed to avoid section-to-section mismatch and to confidently link mass spectrometry data to pathological tissue features with cellular precision. In this case study, the Na^+^ CBS-washed brain tissue was prepared with a 3.2 μg/mm^2^ thick matrix coating, then imaged using 31% TLP (with MALDI-2) before immunofluorescence microscopy. The immunofluorescence microscopy image of leptomeninges in the human frontal cortex, a vasculature region prone to cerebral amyloid angiopathy, is displayed in Figure 2b. Markers were chosen to label vasculature (Collagen IV antibody) and highlight vascular amyloid deposition (overlapping Collagen IV antibody and thiazine red dye), a hallmark feature of cerebral amyloid angiopathy. The advantages of multimodal experimental design are evident in Figure 2c, where TAGs were detected in Alzheimer’s-affected brain tissue and linked to these structures marked by immunofluorescence. Interestingly, the spatial distribution of TAG 50:0 (*m*/*z* 857.756, −1.57 ppm), a lipid associated with Alzheimer’s disease, was found to be localized specifically to vascular regions (Figure 2c).^1^ Qualitatively, both the H&E and immunofluorescence stains (Figures S2 & Figure 2b, respectively) revealed no sign of laser ablation craters or decreased antibody binding, reinforcing the multimodal compatibility of this method.

## Conclusion

In this work, we systematically assessed the effects of salt doping, laser power, and MALDI-2 postionization to enhance the detection of TAGs in IMS experiments, while explicitly considering compatibility with sequential-acquisition single-section multimodal workflows. These studies demonstrate that Na^+^ CBS doping, when paired with MALDI-2, offers a tunable strategy to increase TAG signal intensity and molecular coverage, even at moderate primary laser powers (TLPs ≤31%). Critically, it is now possible to improve sensitivity without inducing significant laser-related tissue damage, preserving the integrity of the tissue required for sequential microscopy experiments as part of multimodal workflows. Beyond these specific findings, this work addresses a broader analytical challenge in the field of how to improve sensitivity and molecular coverage for molecular classes that suffer from poor ionization efficiency without requiring a separate targeted assay. By demonstrating that salt-enhanced MALDI-2 can overcome these barriers using accessible workflows, this study expands the chemical space applicable to MALDI imaging and provides a protocol for routine TAG analysis.

Having reliable methods to fine-tune sensitivity without excessive laser fluence is critical for multimodal studies. As high-dimensional approaches such as multiplexed immunofluorescence microscopy emerge to define cellular organization within tissues, there is a growing need to incorporate molecular readouts that can be integrated across spatial scales. Here, the ability to map TAG distributions on the same tissue section as immunofluorescence and histological stains enables direct spatial registration and mapping of neutral lipid species to defined cellular and pathological features. In the Alzheimer’s disease case study, this approach revealed spatial localization of TAGs to vascular regions associated with cerebral amyloid angiopathy pathology, illustrating the potential of this method to uncover biologically meaningful spatiomolecular signatures inaccessible through bulk analyses.

In conclusion, this work establishes salt-enhanced MALDI-2 as an effective and widely applicable method for neutral lipid imaging that balances sensitivity, molecular coverage, and multimodal compatibility. By enabling robust TAG imaging alongside complementary microscopy, this approach facilitates direct integration of neutral lipid profiles with in situ cellular phenotypes, expanding the molecular classes that can be mapped by IMS as part of multimodal, systems-level studies of human disease.

## Supplementary Material





## Notes

Table of all annotated TAGs. (XLSX)
Supplementary Content. Extended materials and methods. Table of instrumental parameters for MALDI-2 IMS. Staining microscopy of imaged human brain. Bar plot of alternative S/N cutoffs for TAG annotations. TAG standard spectra. Matrix density comparison with tissue homogenate.(PDF)
