# Supplementary material for: Optimized Sodium-Enhanced MALDI‑2 Enables Multimodal Imaging of Triacylglycerols
Source: J Am Soc Mass Spectrom. 2026 Aug 25;37(10):2618–22. doi: 10.1021/jasms.6c00227 (PMC13651605; doi:10.1021/jasms.6c00227)
Supplement: Supplementary file 1 [file js6c00227_si_002.pdf]

Supplemental Materials

Optimized Sodium-Enhanced MALDI-2 Enables Multimodal Imaging of Triacylglycerols

Kameron R. Molloy[1, 2], Cody R. Marshall[2, 3], Martin Dufresne[2, 4], Lukasz G. Migas[2,5], Matthew Schrag[6, 7, 8], Raf Van de Plas[2, 5, 9], Jeffrey M. Spraggins[1, 2, 3, 4, 9, 10*]

[1]Department of Chemistry, Vanderbilt University, Nashville, TN 37235

[2]Mass Spectrometry Research Center, Vanderbilt University, Nashville, TN 37235

[3]Chemical and Physical Biology Program, Vanderbilt University School of Medicine, Nashville, TN 37240

[4]Department of Cell and Developmental Biology, Vanderbilt University, Nashville, TN 37232

[5]Delft Center for Systems and Control, Delft University of Technology, 2628 Delft, Netherlands

[6]Department of Neurology, Vanderbilt University School of Medicine, Nashville, TN 37232

[7]Vanderbilt Brain Institute, Vanderbilt University, Nashville, TN 37240

[8]Vanderbilt Memory and Alzheimer's Center, Vanderbilt University Medical Center, Nashville, TN 37232

[9]Department of Biochemistry, Vanderbilt University, Nashville, TN 37205

[10]Department of Pathology, Microbiology and Immunology, Vanderbilt University Medical Center, Nashville, TN 37205

*Corresponding Author: Jeffrey M. Spraggins, phone: +1 (615) 343-7333, email: jeff.spraggins@vanderbilt.edu

Extended Methods

Materials

HPLC-grade acetone, ethanol (EtOH), methanol (MeOH), isopropyl alcohol (IPA), acetonitrile (ACN), methyl tert-butyl ether (MTBE), and formic acid were acquired from Fisher Scientific (Pittsburgh, PA, USA). Tris-buffered saline (TBS), 0.05% Tween-20, fish gelatin, 100 mM glycine, 0.1% Triton X-100, and bovine serum albumin were purchased from Sigma-Aldrich (St. Louis, MO). Sucrose solution, ammonium formate (AmF), sodium and potassium carbonate, bicarbonate, and acetate were purchased from Sigma-Aldrich (St. Louis, MO, USA). Paraformaldehyde (PFA) and 20 mM Hoechst 33342 were obtained from Thermo Scientific (Waltham, MA). The matrix, 2', 5'-dihydroxyacetophenone acid (DHA), was purchased from TCI America (Portland, OR). Commercial antibodies for alpha smooth muscle actin (αSMA) (Abcam, Cambridge, UK; AB124964), collagen IV (NeoBiotechnologies, Union City, CA; M3F7), AlexaFluor 750 goat anti-rabbit (Abcam, AB175735), and Cy5 goat anti-mouse (Abcam, AB6563) were purchased. Normal donkey serum (AB7475) was

also acquired from Abcam (Cambridge, UK), while thiazine red was purchased from Chemsavers (Bluefield, VA).

MALDI IMS Sample Preparation

Sodium carbonate buffer (Na^+ CBS) and 150 mM AmF were made, then Na^+ CBS was diluted by 2.5x, and both were stored at 4°C. Na^+ CBS, a carbonate buffer solution spiked with acetate, as outlined by Dufresne et al., and final concentrations of each component are discussed by Molloy et al.[5, 7]

To prepare the multi-tissue homogenate, fresh frozen tissues including rat brain, rat liver (BioIVT, Hicksville, NY, USA), rabbit testes, and mature rabbit adrenal gland (Pel-Freez Biologicals, Rogers, AR) were cryosectioned using a Leica CM3050 cryostat (Leica Microsystems GmbH, Wetzlar, Germany) at 50 µm thickness and 'scrolls' were collected into 1.5 mL GATOR™ snap-cap tubes prefilled with zirconium oxide beads (Next Advance, Inc., Troy, NY, USA). ~350 mg of each tissue was collected into the same tube before homogenizing with a Bullet Blender Tissue Homogenizer (Next Advance, Inc., Troy, NY, USA). A plastic syringe was used to move the homogenate from the bead tube onto a stainless steel plate sitting over dry ice to freeze. The frozen homogenate pellets were then stored at -80°C.

A tissue block from the frontal cortex of a human brain donor with severe Alzheimer's disease and severe cerebral amyloid angiopathy (provided by the brain bank in Dr. Schrag's laboratory - Vanderbilt University, Nashville, TN, USA) was frozen in liquid nitrogen, embedded with 15% fish gelatin, and then stored at -80°C.

Homogenate and human brain tissues were cryosectioned at 10 µm thickness and thaw-mounted onto indium tin oxide (ITO) coated glass slides (Delta Technology, Loveland, CO). Mounted tissue sections were submerged in either AmF or Na^+ CBS three times for 45 seconds, then set upright to dry in a vacuum desiccator for 10 min. Next, 2',5'-dihydroxyacetophenone acid (1.6 or 3.1 ± 0.2 $\mu g/mm^2$) was prepared in acetone and applied to all slides using an in-house built prototype sublimation device akin to the commercial HTX SubliMATE (HTX Technologies, LLC, Chapel Hill, NC). Matrix density was calculated by weighing a slide before and after sublimation, then dividing by the covered surface area. This was repeated four times, then averaged to obtain the reported values. During sublimation, the cold finger was cooled using dry ice and acetone. Slides were allowed to cool for 5 minutes before heating to 150°C for 10 min at 10 mTorr. Next, dry ice was removed, dropping the temperature to 100°C before adding a 120°C hot puck to bring the chamber to room temperature, before breaking vacuum.

To evaluate in-source decay, a TAG (54:2) standard (Cayman Chemical, Ann Arbor, MI) was dissolved in MTBE to a final concentration of 10 mM then spotted onto an MTP 384 ground steel target plate (Bruker Daltonik, Bremen, Germany) as follows. First, DHA (0.50 µL) was spotted on the target plate. Once dry, the standard solution (0.25 µL) was applied, followed by 0.50 µL salt solution, then 0.50 µL DHA. The layered spot was allowed to dry at room temperature.

MALDI and MALDI-2 IMS

Homogenate and brain tissue, and standard spot MALDI IMS analyses were performed on a timsTOF fleX MALDI-2 mass spectrometer equipped with a stacked ring ion guide (SRIG) mobility funnel (Bruker Daltonik, Bremen, Germany). Homogenate and brain tissue IMS data were acquired with a 20 μm and 15 μm pitch, respectively. Across all studies, data were collected in positive ion mode with a source temperature of 50°C, a TIMS in pressure of 2.68 ± 0.03 mbar, and an *m/z* range from 200-1300. All homogenate sections were imaged with and without MALDI-2 at five different MALDI laser powers: (21%, 26%, 31%, 36%, and 41%). Total laser powers (TLPs) were calculated by multiplying the laser attenuator range by the laser percentage chosen in the timsControl interface, then adding the global attenuator offset. For each increase in laser power, a 5% global attenuator boost was added, resulting in an ~8% increase in the total laser power applied. For MALDI-2 analyses, images were acquired with 10 shots per pixel, and MALDI analyses were performed using 25 shots per pixel. These parameters were chosen to ensure optimized performance for MALDI and MALDI-2 analyses individually. A comprehensive table of instrument parameters is provided in Table S1.

Complementary Microscopy

Microscopy images were collected before applying matrix with DAPI, eGFP, Cy5, and DSRed fluorescent filters on a Zeiss AxioScan.Z1 fluorescence slide scanner (Carl Zeiss Microscopy GmbH, Oberkochen, Germany). After performing MALDI IMS, microscopy images were collected with eGFP and brightfield filters, without removing the MALDI matrix. Next, the brain tissue was prepared for immunofluorescence staining by first removing the matrix layer with a gradient of EtOH washes (70% to 90% EtOH). Then, the tissue was fixed by submerging in 1% PFA solution for 10 minutes before treating with a 10% sucrose solution (w/v) for 1 hour. After this, the tissue was washed in 100mM glycine/1xTBS/0.1% Triton X-100 buffer for 30 minutes, then blocked with 10% normal donkey serum in 1xTBS/0.1% bovine serum albumin at 37°C for 1 hour. The tissue was then stained with thiazine red and Hoechst nuclear stain with primary antibodies for Collagen IV and αSMA before washing with 1xTBS/0.05% Tween-20. Finally, 50% glycerol was used for coverslipping. Immunofluorescence microscopy images were collected with DAPI, eGFP, Cy5, Cy7, and DSRed fluorescent filters. Following this, the coverslip was removed by dipping in 1xTBS, then the samples were stained with H&E, and a new coverslip was applied with cytoseal.

Data Analysis

Ion images were visualized, and immunofluorescence images were co-registered to IMS data in SCiLS (Bruker Daltonik, Bremen, Germany). Mass spectra were normalized, aligned, and annotated using in-house developed IMS data processing and annotation software. A 5 ppm error threshold was used to make tentative peak identifications, and annotations were only included if the lipid was detected in all three replicates. Intensities within one mass bin of the theoretical *m/z* values for each TAG species were extracted to track the prevalence of each species across imaging experiments. Error bars represent the standard error of the mean across all detected TAG species within each ionization condition (n = 174; 29 TAGs with 2 adducts and 3 replicates). The smallest dataset contained 1,931 pixels; therefore, all larger datasets were deterministically subsampled to 1,931 pixels using a distributed frame-selection approach. Selected pixels were spaced across the full frame list and trimmed to the target count, ensuring that intensity comparisons were performed using the same number of pixels per dataset. Next, all datasets were normalized to total ion current (TIC), and to

account for run-to-run variation, inter-dataset normalization was performed by rescaling the normalization factors from all datasets. Intensities were calculated by integrating the area within one *m/z* bin of each annotated peak. Finally, signal-to-noise ratios were calculated and used to compare which TAG species were detected under every condition set. Results using varying signal-to-noise cutoffs are shown in **Fig S1.**

Table S1. Instrument parameters used for MALDI and MALDI-2 IMS analyses.

**Transfer**	
MALDI Plate Offset	50 V
Funnel 1 RF	500 Vpp
isCID Energy	0 eV
Funnel 2 RF	400 Vpp
Multipole RF	600 Vpp
**Collision Cell**	
Collision Energy	10 eV
Collision RF	2000 Vpp
**Quadrupole**	
Ion Energy	5 eV
**Focus Pre TOF**	
Transfer Time	80 µs
PrePulse Storage	16 µs

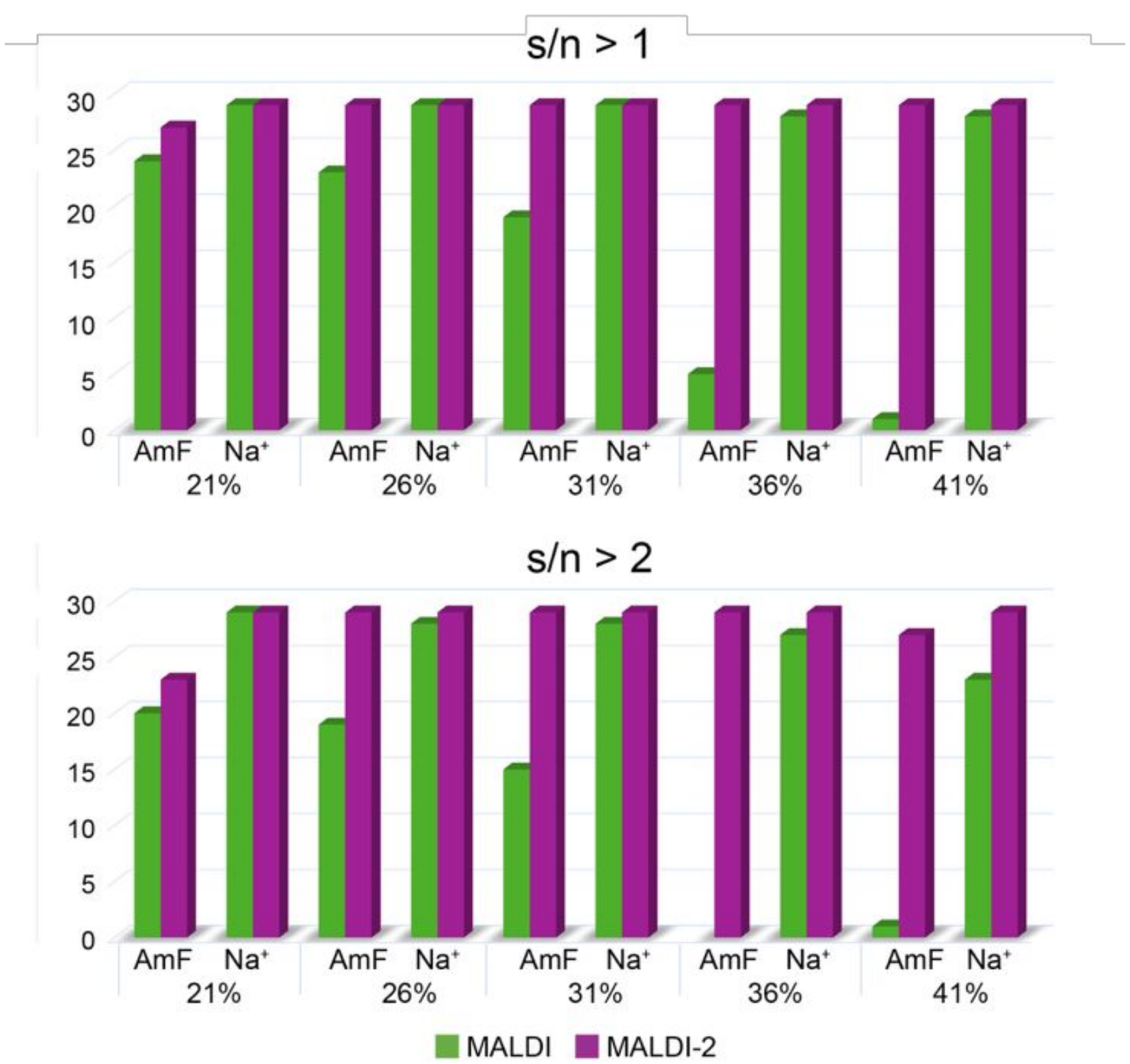

Figure S1. The number of TAGs annotated using alternative signal-to-noise thresholds. S/N > 1 (top) and S/N > 2 (bottom).

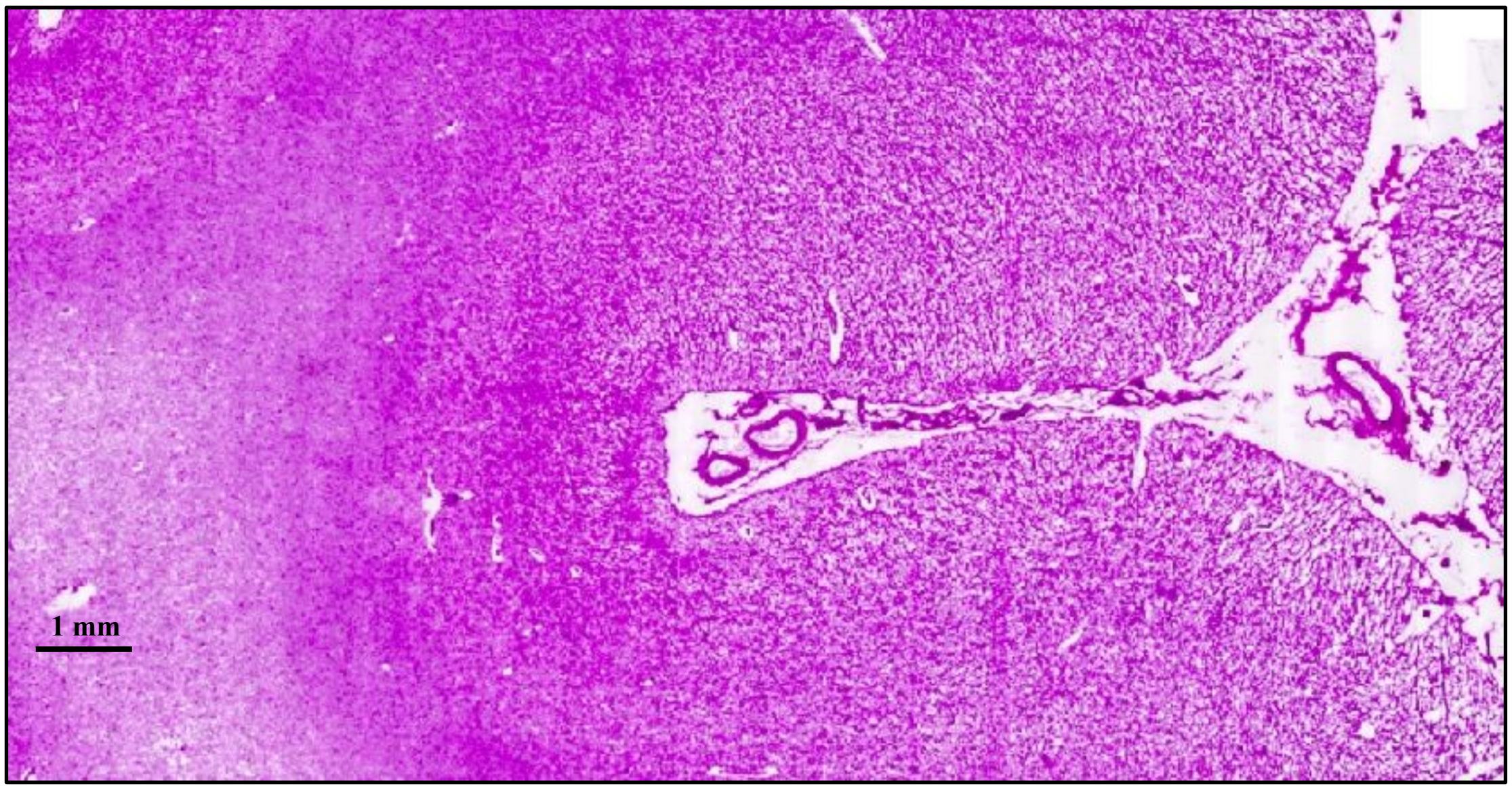

Figure S2. H&E-stained brightfield microscopy image of the MALDI imaging region from a human brain affected by CAA and AD, which was analyzed using 31% TLP for the MALDI experiment.

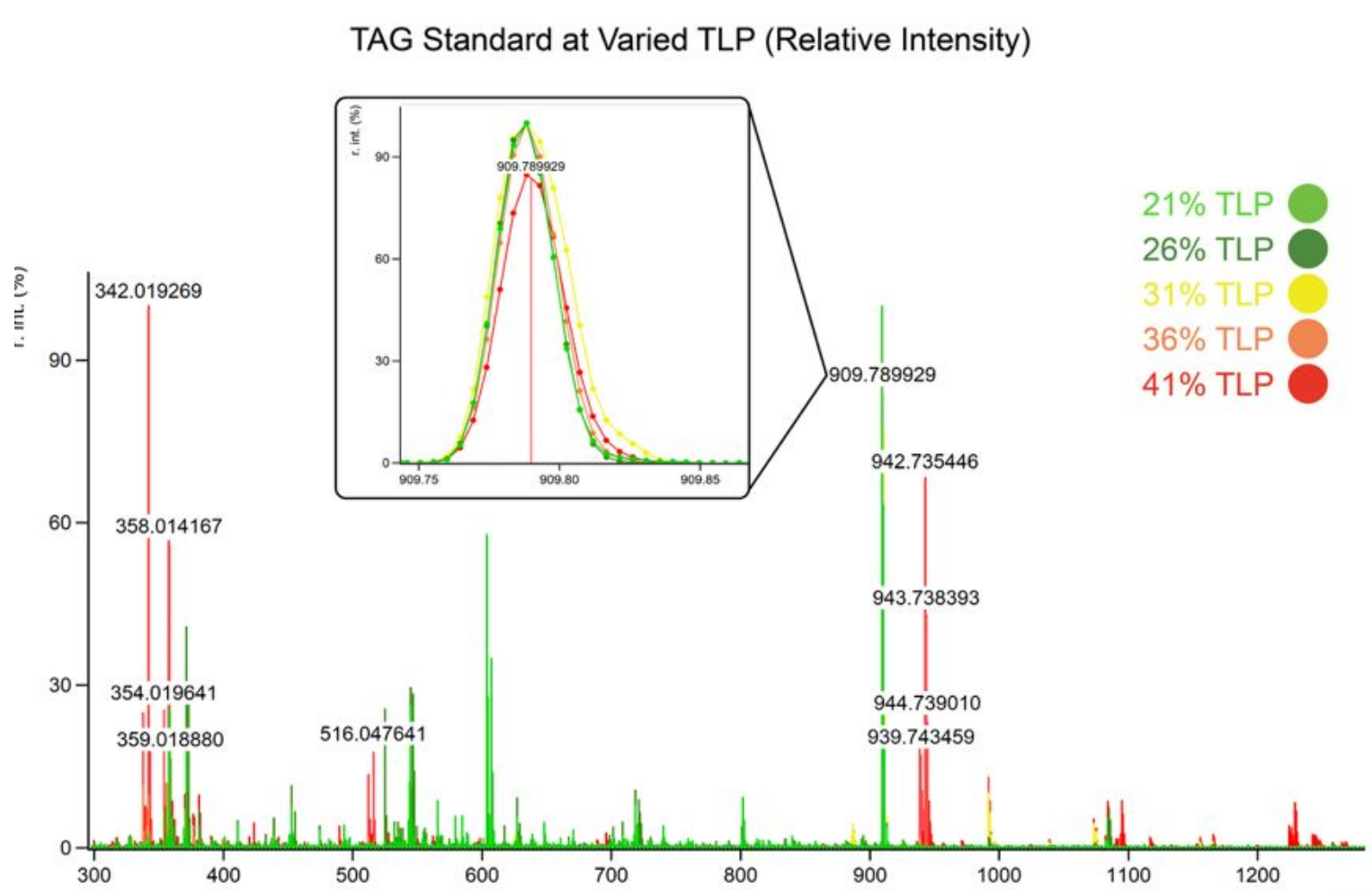

Figure S3. TAG 54:2, *m/z* 909.790 standard evaluated for in-source decay. The standard was spotted with Na^+ CBS and DHA, then imaged at the five tested TLPs with MALDI-2. Each spectrum is the average of spectra collected at five locations, each with 10 laser shots. Relative intensities of the corresponding spectra were visualized. Zoomed regions show the parent ion as TLP increases.

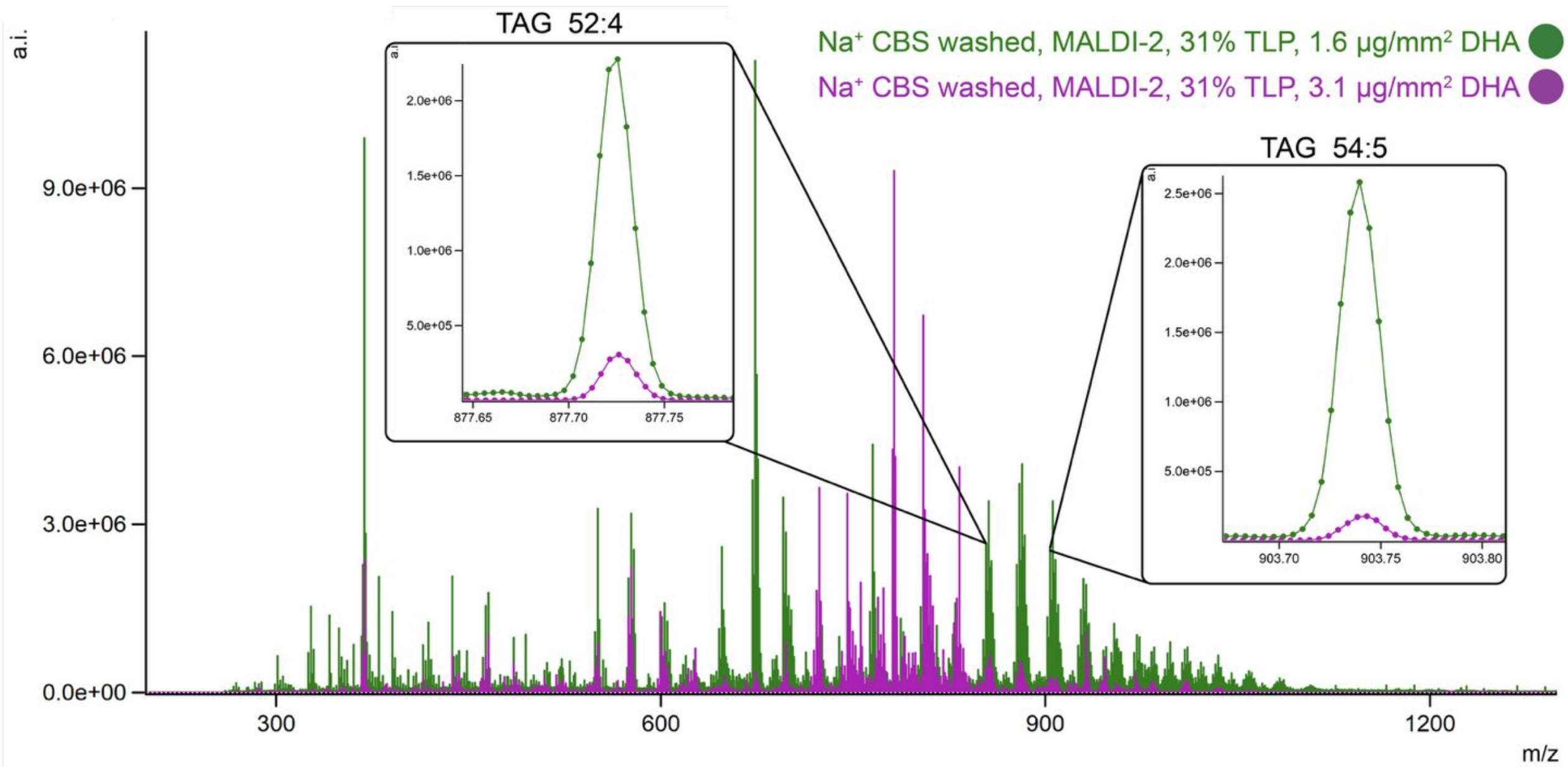

Figure S4. Spectral comparison of a multi-tissue homogenate imaged with MALDI-2 at 31% TLP with two different matrix concentrations. Increased matrix reveals a decrease in signal intensity among the TAGs.
